# The Relationship between Associative Learning, Transfer Generalization, and Homocysteine Levels in Mild Cognitive Impairment

**DOI:** 10.1371/journal.pone.0046496

**Published:** 2012-09-28

**Authors:** Ahmed A. Moustafa, Doaa H. Hewedi, Abeer M. Eissa, Catherine E. Myers, Hisham A. Sadek

**Affiliations:** 1 School of Social Sciences and Psychology & Marcs Institute for Brain and Behaviour, University of Western Sydney, Sydney, New South Wales, Australia; 2 Psychogeriatric Research Center, Department of Psychiatry, School of Medicine, Ain Shams University, Cairo, Egypt; 3 Department of Psychology, Rutgers University-Newark, Newark, New Jersey, United States of America; 4 NeuroBehavioral Research Laboratory, Department of Veterans Affairs-New Jersey Health Care System, East Orange, New Jersey, United States of America; University Of São Paulo, Brazil

## Abstract

Previous studies have shown that high total homocysteine levels are associated with Alzheimer's disease (AD) and mild cognitive impairment (MCI). In this study, we test the relationship between cognitive function and total homocysteine levels in healthy subjects (Global Dementia Rating, CDR = 0) and individuals with MCI (CDR = 0.5). We have used a cognitive task that tests learning and generalization of rules, processes that have been previously shown to rely on the integrity of the striatal and hippocampal regions, respectively. We found that total homocysteine levels are higher in MCI individuals than in healthy controls. Unlike what we expected, we found no difference between MCI subjects and healthy controls in learning and generalization. We conducted further analysis after diving MCI subjects in two groups, depending on their Global Deterioration Scale (GDS) scores: individuals with very mild cognitive decline (vMCD, GDS = 2) and mild cognitive decline (MCD, GDS = 3). There was no difference among the two MCI and healthy control groups in learning performance. However, we found that individuals with MCD make more generalization errors than healthy controls and individuals with vMCD. We found no difference in the number of generalization errors between healthy controls and MCI individuals with vMCD. In addition, interestingly, we found that total homocysteine levels correlate positively with generalization errors, but not with learning errors. Our results are in agreement with prior results showing a link between hippocampal function, generalization performance, and total homocysteine levels. Importantly, our study is perhaps among the first to test the relationship between learning (and generalization) of rules and homocysteine levels in healthy controls and individuals with MCI.

## Introduction

Increasing number of studies has shown that homocysteine (a compound found in the blood) is associated with mild cognitive impairment (MCI), dementia, and Alzheimer's disease (AD) [1], [2], [3], [4], [5]. Based on these studies, in this project, we test the relationship between homocysteine levels and cognitive function in healthy controls and individuals with MCI. Unlike prior research (which has mostly used questionnaires), here, we study the relationship between homocysteine levels and learning and generalization of rules.

Below, we discuss the neural and behavioral correlates of homocysteine and MCI. Then, we review prior studies on the role of the hippocampus and basal ganglia in learning and generalization.

### Homocysteine: neural and behavioral correlates

Previous studies have shown that total serum homocysteine levels are associated with various brain disorders [1], [2], including Alzheimer's disease [3], [4], [5], [6], [7], MCI [3], [8], stroke [9], and movement disorders [10]. Interestingly, it was found that baseline measures of homocysteine levels in AD patients and healthy subjects predict subsequent cognitive decline, as measured by the Cambridge cognitive testing battery (CAMCOG) [11], mini-mental state examination (MMSE), and the cognitive subscale of the Alzheimer's disease Assessment Tool (ADAS-Cog) [12]. Along the same lines, studies suggest that low levels of homocysteine in individuals with MCI are protective against conversion to dementia [13].

Hyperhomocysteinemia is a condition associated with increased levels of homocysteine in the blood. Interestingly, various studies have shown that hyperhomocysteinemia is relatively more common in AD patients than in controls [5]. Recently, Pirchl, Ullrich, and Humpel [14] have found hyperhomocysteinemia is associated with a reduction of cortical acetylcholine in rats. Abnormal acetylcholine levels has been linked to AD and MCI [15], [16], [17], [18], [19]; it is possible that increased levels of homocysteine leads to a reduction in acetylcholine levels, and thus cause memory/cognitive symptoms of AD. In addition, recent research has also shown that lowering homocysteine levels is neuroprotective in MCI [20]. Along the same lines, studies in rats found that hyperhomocysteinemia is associated with impaired performance in the Morris water maze task [21], [22], which tests spatial learning and memory, and was found to rely on the hippocampus [23], [24]. Importantly, recent clinical trials are investigating the therapeutic efficacy of homocysteine-lowering drugs in AD patients (see www.clinicaltrials.gov). These studies and clinical trials stress the importance of understanding the relationship between homocysteine and cognition.

Studies measuring total homocysteine levels in healthy individuals (especially in elderly populations) have shown that homocysteine plays an important role in cognitive processes [25], [26], [27], [28], [29], [30]. Specifically, Nurk et al. [25] found that increased homocysteine levels in healthy individuals is associated with impaired episodic memory performance. Along the same lines, van de Kommer, Dik, Comijs, Jonker, and Deeg [31] reported that higher homocysteine levels are associated with slow information processing speed and fluid intelligence in healthy adults. Also, Garcia, Haron, Pulman, Hua, and Freedman [32] have shown that higher levels of homocysteine are correlated with impaired performance in the Stroop test. Specifically, homocysteine was shown to be involved in episodic memory [3], [33], spatial learning [14], reversal learning [34], [35], and executive function [33]. On the other hand, studies have suggested homocysteine is perhaps not associated with other cognitive processes, including working memory and attention [33], although other studies reported that lowering homocysteine levels enhance working memory [36].

The exact function of homocysteine is not known. However, prior studies have shown that homocysteine acts on various brain regions, including the hippocampus [37], [38], [39], cortex [39], and the basal ganglia [40]. Higher homocysteine levels lead to atrophy in the frontal, parietal, and temporal areas [41]. Also, various studies have suggested that homocysteine might regulate the function of other neuromodulators, such as acetylcholine [37] and dopamine/serotonin [22]. Specifically, Gao et al. [22] have reported that rats with hyperhomocysteinemia have lower level of dopamine and serotonin in the cortex than control rats. Other studies suggest that homocysteine regulates synaptic plasticity in the hippocampus [34], [35]. These prior studies suggest that homocysteine has multiple functions in the brain.

### Mild cognitive impairment

Mild cognitive impairment (MCI) is a state of cognitive decline greater than that expected for an individual's age and education level, but falling short of dementia [42]. Individuals with MCI are statistically at increased risk to develop AD within the next several years [43], [44], which suggests that MCI may reflect gradual accumulation of AD pathology, though at a level not yet sufficient to cause catastrophic decline in cognitive function. This is particularly true of the MCI subgroup with memory loss as a predominant syndrome, a condition termed amnestic MCI [45]. Studies suggest that individuals with MCI tend to progress to AD at a rate of 10–15% per year [46], [47], [48], and many researchers consider MCI to be an early or prodromal form of AD.

### The role of the basal ganglia and hippocampus in learning and generalization

Prior research has shown that learning of rules and generalization of these rules in new contexts are subserved by different brain systems.

Several studies have shown that the basal ganglia is involved in learning from corrective feedback [49]. In feedback learning tasks, subjects learn to associate the presentation of different stimuli with different responses, based on corrective feedback. For example, animal literature has also shown that striatal cells show increased activity during stimulus-response learning [50]. Also, fMRI studies have shown basal ganglia is active in during feedback learning tasks [49], [51]. Along the same lines, patients with Parkinson's disease patients (disease associated with basal ganglia dysfunction) show impairment at the learning phase of the same task used here [52]. Using various learning tasks, studies show that dopamine medications and agents impair learning in both Parkinson's disease patients [53], [54], possibly by affecting the basal ganglia structure. Recent reviews by Seger and colleagues provide extensive discussion on the role of the basal ganglia in learning [55], [56].

The hippocampus participates in the generalization of learned rules [57], [58], [59], [60]. Patients with hippocampal damage are impaired at retrieving information when study and test conditions are different [61], [62], [63]. Other research has shown that the hippocampus is important for the generalization of learned rules in various paradigms, including transitive inference, sensory preconditioning, and acquired equivalence (which we describe below). For example, several studies have shown that the hippocampus is involved in transitive inference, in which subjects learn to deduce new information from previously learned rules (e.g., if A>B & B>C, we conclude that A>C) [64], [65]. Using fMRI, Shohamy and colleagues [60] have found that the hippocampus is active while subjects performing the sensory preconditioning task, in which if a subject is first given unreinforced trials with stimuli A and B presented together as a compound cue (AB -), then training the subject that A (alone) predicts a certain outcome will lead some of this association to be transferred to B—that is, subjects also learn that B predicts the same outcome as A [66]. Shohamy and colleagues found that the hippocampus is important for generalization of rules in this paradigm.

Another paradigm that involves generalization of rules is the acquired equivalence task. In this task, stimuli become equivalent when they are associated with the same outcome [67]. For example, if cue A is associated with outcome C, and cue B is also associated with the same outcome C, subjects learn that A and B are associated (which is a gernalization from previously learned rules). Research at our lab has shown that hippocampal atrophy interferes with generalization performance in the acquired equivalence task [62]. Similarly, rats trained to choose among two odors (A or B in some trials, or C or D in other trials), based on reinforcement given to the choice of one of them (A in AB trials, or C in CD trials) would generalize well to novel pairing of familiar odors, that is, they will choose A in AD trials, and C in CB trials. However, animals with hippocampal dysfunction performed at chance on these novel pairings [57]. The learning-and-generalization task used in our study is an example of such generalization task and is similar to the animal study used by Eichenbaum and colleagues [57], in that subjects learn to generalize to previously learned rules (see description below). We have recently found that the hippocampus is active in elderly but not in MCI subjects while performing learning-and-generalization tasks [68]. These prior data show that the hippocampus participates in generalization of learned rules in various experimental paradigms.

In sum, prior studies suggest that learning and generalization of learned rules are subserved by different brain systems, namely the basal ganglia and hippocampus. In the current study, we test if these cognitive processes are affected by homocysteine levels in healthy controls and individuals with MCI.

## Methods

Below, we describe details on subject recruitment, neuropsychological assessment, the measures of homocysteine levels from blood samples collected from all subjects, and the computerized learning-and-generalization task.

### Subjects

All subjects were screened based on self-reports for medical or psychiatric history, including presence of depression, multiple sclerosis, aphasia, and seizure/epilepsy. We also excluded subjects who showed signs of dementia. We recruited 59 individuals from the department of Psychiatry, Ain Shams University. All subjects signed statements of informed consent before testing was initiated. Research conformed to guidelines for protection of human subjects established by Ain Shams University's School of Medicine. Ethics committee at Ain Shams University's School of Medicine approved this study.

### Neuropsychological Assessment

We screened subjects who complained of subjective memory impairment using the mini-mental status examination (MMSE) which is a screening test to prove that subjects are not suffering from definite memory impairment [69]. We excluded 4 subjects who had low MMSE scores (less than 24) or appear to show signs of dementia as observed by the neurologists (D.H.H and A.M.E).

We then conducted the Clinical Dementia Rating (CDR) scale, which was designed to identify the degree and severity of dementia in human subjects [44], [70], [71]. The CDR evaluates problem solving abilities, orientation in time and place, personal care skills, home activities, among others. The CDR scores range from 0 to 3, and indicate no dementia (CDR = 0), mild cognitive impairment (MCI; CDR = 0.5), mild dementia (CDR = 1), moderate dementia (CDR = 2) or severe dementia (CDR = 3). As in previous studies, all controls in our study had Clinical Dementia Rating (CDR) of 0, while individuals with MCI have CDR of 0.5 [70], [72].

In addition, we assessed all subjects for cognitive impairments using the Global Deterioration Scale (GDS) [73], [74] which ranks individuals according to a 7-point scale. The GDS 1 rating is given to an individual with no memory impairment. The GDS 2 rating is given to an individual who is functionally unimpaired but with subjective complaints of mild forgetfulness that is not recognized by family members or coworkers and for which there is no clinical evidence. The GDS 2 score refers to a condition known as very mild cognitive decline (vMCD). The GDS 3 rating is given to an individual with subtle functional deficits, revealed with extensive clinical interview. Whereas GDS 3 rating does not indicate dementia, individuals with GDS 3 ratings are at heightened risk to subsequently develop AD, compared with individuals given GDS ratings of 1 and 2 [43], [75]. The GDS 3 score refers to a condition known as mild cognitive decline (MCD). GDS rating of 4 and higher indicate dementia with increasingly severe cognitive and functional impairments; GDS 4 is often considered indicative of mild AD. To be included in the current study, individuals were required to have ratings of GDS 3 or lower, indicative of nondemented clinical status. In total, we excluded 3 subjects who had dementia (as measured by CDR or GDS).Overall, subjects in the current study had an average GDS rating of 1.93 (SD = 0.68).

The final sample consisted of 52 subjects who scored at or above age-appropriate norms on standardized neuropsychological tests. These 52 participants were administered the learning-and-generalization task. We have excluded another 3 subjects who did not pass the criterion in the learning phase of the task (see description below). Results for the 49 participants are shown in Table 1.

**Table 1:** Demographic information of healthy controls and individuals with MCI in the learning-and-generalization study.

Group	N	n filt	Sex ratio (m∶f)	Age	Years Education	CDR	GDS	MMSE
Controls	26	24	11∶13	66.3 (5.18)	13.1 (1.34)	0	1.4 (0.51)	27.7 (1.45)
MCI	26	25	10∶15	69.1 (6.42)	12.7 (1.56)	0.5	2.4 (0.50)	26.2 (1.72)

Abbreviation: n is number of subjects we tested; n filt is number of subjects after filtering out subjects who did not learn the task; CDR is clinical dementia rating; GDS is global deterioration scale; MMSE is mini mental status examination.

### Homocysteine levels

We have collected blood samples from all subjects who passed the neuropsychological screening tests mentioned above (N = 52) to measure plasma homocysteine levels using the Homocysteine Enzyme Immunoassay (EIA) method, as outlined in prior studies [76]. This is an enzyme immunoassay for the determination of homocysteine in blood. Specifically, in all healthy controls and MCI individuals, venous blood samples (maximum 2 cm^3^) were withdrawn and put in EDTA anticoagulated tubes to prevent blood clotting. All blood samples were put on ice immediately after drawing for up to 6 hours prior to separation of plasma by centrifugation. Reagents were added to the plasma, which was then kept at room temperature (18–25°C) to avoid destruction of enzymes. In prior studies, higher homocysteine levels have been associated with dementia and cognitive impairment [5], [30], [77]. Here, we tested whether homocysteine levels correlate with performance in the learning-and-generalization task.

### Cognitive task: Learning-and-Generalization

Here we describe details of the learning-and-generalization task. Testing took place in a quiet room at Ain Shams's School of Medicine, with the subject seated in front of a Macintosh MacBook laptop computer with color screen. The keyboard was masked except for two keys, labeled “LEFT” and “RIGHT” which the subject used to enter responses.

**Figure 1:**
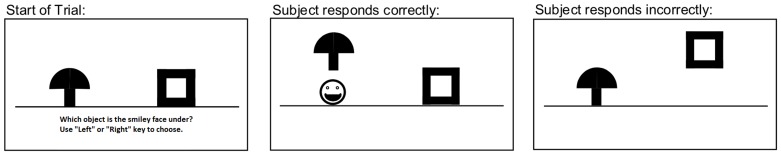
Screen events on a sample trial of phase 1. (A) At start of trial, two objects appear, differing in color or shape but not both. (B) The participant chooses one object and that object is raised; if the choice was correct, a smiley face is revealed underneath. C) If incorrect, there is no smiley face.

The task has two phases: learning and generalization. The learning phase of the task consists of an eight-pair concurrent discrimination. This is an incrementally-acquired, feedback-based learning task in which subjects are to learn, via feedback, which object is correct, and they are given no information about the correct object ahead of time. On each trial, two colored shapes appeared, approximately 1″ in height on the screen and set about 3″ apart (approximately 1.5 degrees of visual angle, at normal viewing distance). The subject was instructed to press the left or right key to choose one object. The chosen object was raised and, if the choice was correct, a smiley face was revealed underneath (see Figure 1). There was no limit on response time, and there was an interval of approximately one second between subject response and start of the next trial, allowing the subject to view the discrimination pair together with feedback (presence or absence of the desired smiley face icon).

Within each object pair, the same object was always rewarded. For four of the discrimination pairs, objects differed in shape but not color (e.g. brown mushroom vs. brown frame); for the remaining four pairs, objects differed in color but not shape (e.g. red cat's-eye vs. yellow cat's-eye). Thus, within each pair, one dimension (color or shape) was relevant with respect to predicting the location of the smiley face, and one dimension was irrelevant.

Trials were organized into blocks, each containing 16 trials: one presentation of each discrimination pair in each possible left-right ordering. Trials in a block occurred in a pseudorandom but fixed order. Phase 1 continued until the subject reached a criterion of 16 consecutive correct responses, or for a maximum of 96 trials (6 blocks).

After the learning phase, the generalization phase began without any warning to the subject. The screen events were identical to the learning phase except that the discrimination pairs were altered so that the relevant features remained constant but the irrelevant features were altered. Thus, for example, the learning phase discrimination in which a brown mushroom was rewarded over a brown frame became in the generalization phase a discrimination in which a green mushroom was rewarded over a green frame. Similarly, the learning phase discrimination in which a red cat's-eye was rewarded over a yellow cat's-eye became in the generalization phase a red/yellow discrimination involving a new shape. Individuals who had previously solved the learning phase by basing associations on the relevant features (mushroom beats frame and red beats yellow) could perform perfectly in the generalization phase, since the relevant features are still predictive. By contrast, individuals who had approached the learning phase by learning to respond to whole objects (brown-mushroom beats brown-frame) should perform poorly in the generalization phase where there are novel objects (green-mushroom and green-frame).

Generalization phase trials were organized into blocks of 16 trials, one trial with each discrimination pair in each possible left-right ordering, in a pseudorandom but fixed order. The generalization phase continued until the participant reached a criterion of 16 consecutive correct responses, or to a maximum of 48 trials (3 blocks). The entire procedure took approximately 15–20 minutes to complete.

## Results

For all analyses, we used SPSS and SAS v8.0 PROC MIXED to examine between-subject differences, using unstructured covariance matrices (which does not make any strong assumptions about the variance and correlation of the data, as do structured covariances).Where indicated, we tested for specific planned contrasts. In these contrasts, the number of degrees of freedom reflects the entire sample, and not just the subjects involved in the particular contrast, because the mixed procedure analyzes between-subject effects, and controls for other variables of interest that apply across all subjects. This procedure uses all of the data to provide a more stable estimate of the error term.

### Healthy controls vs. individuals with mild cognitive impairment

As mentioned above, for the purposes of analyses, subjects were divided into two groups: Healthy controls (CDR = 0) and individuals with MCI (CDR = 0.5).

We found that only one healthy subject finishes the acquisition phase in five blocks (80 trials), while the rest of subjects took all six blocks (96 trials). In the generalization phase, all subjects took all 48 trials. This means that most subjects have done more or less the same number of trials throughout the task.

**Figure 2:**
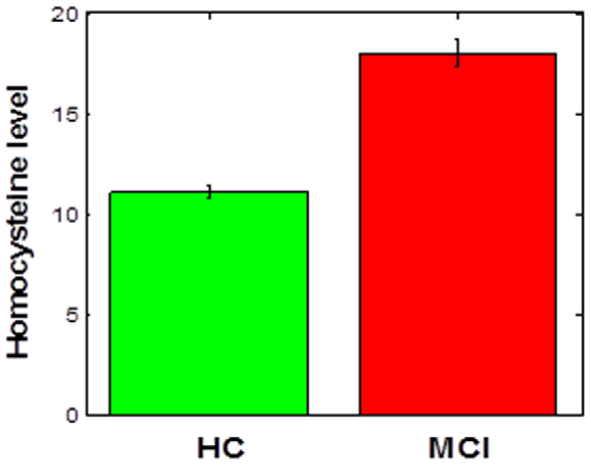
Total homocysteine levels are higher in individuals with MCI than in controls. HC = healthy controls.

**Figure 3:**
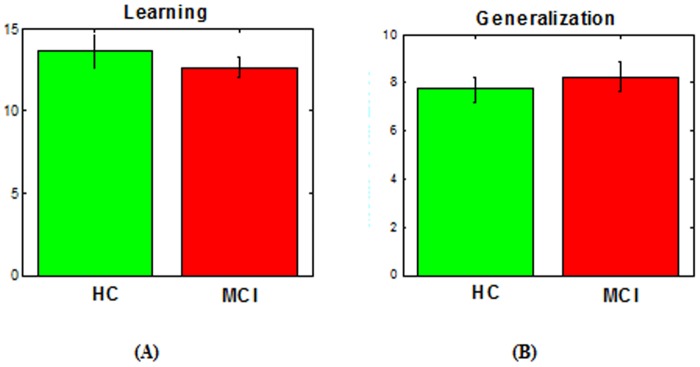
Learning and generalization performance in MCI and healthy control individuals. We have found no difference between MCI and HC either in the number of errors the learning (A) or generalization phase (B).

In addition, we tested if there were any differences among the subjects on (a) homocysteine levels and (b) number of errors in the learning and generalization phases. We found there is a significant effect of group on homocysteine levels (p < 0.001), such as homocysteine levels are significantly higher in individuals with MCI than in healthy controls (Figure 2). However, there was no effect of group on either learning (p>0.2, Figure 3A) or generalization performance (p>0.1, Figure 3B).

**Figure 4:**
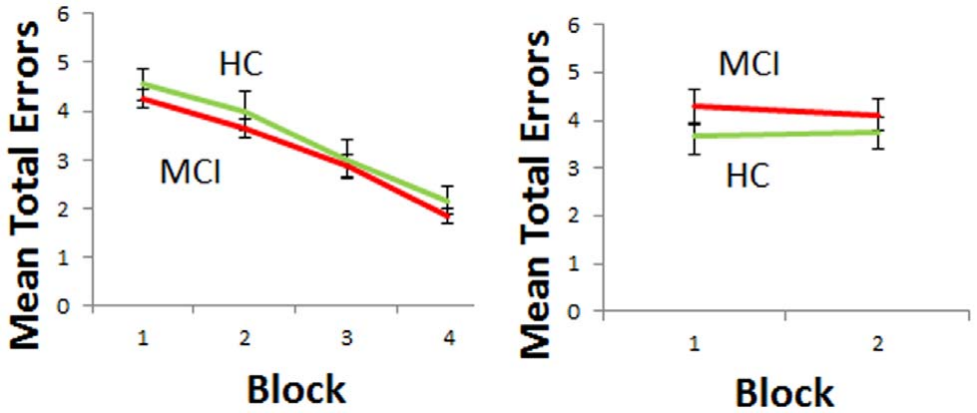
Mean total of errors in the learning and generalization task in MCI and healthy control individuals. We found an effect of block in both groups in the learning but not generalization phase. As in prior analysis, by adding block number as a variable in ANOVA analysis, we found no difference between MCI and HC either in the number of errors the learning (A) or generalization phase (B).

To test for learning effects, we divided performance into different blocks (each is 24 trials). One subject did 80 trials in the acquisition phase (with blocks has 20 trials each). We found that across all subjects, there was an effect of block in the learning (p = .001) but not generalization phase (p = 0.12). Same effects of block was correct for all groups (p's < = 0.04; see Figure 4). In addition, there was no difference between MCI and controls in any of the blocks in acquisition or generalization phases.

**Figure 5:**
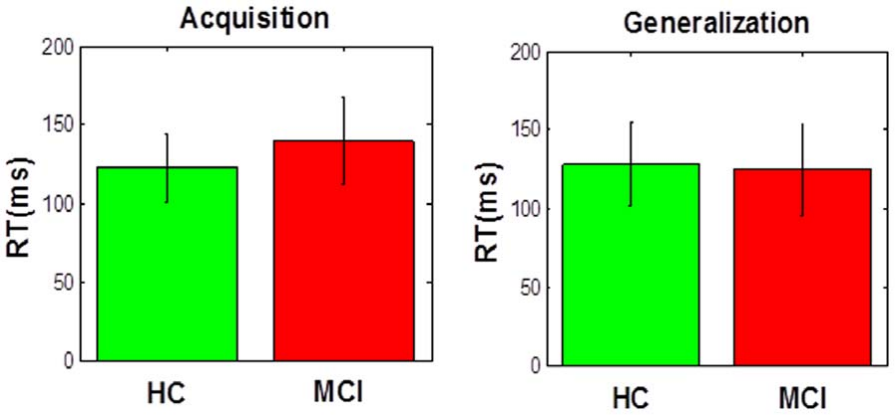
Reaction time in the learning and generalization task in MCI and healthy control individuals. We have found no difference between MCI and HC in RT in the learning (A) or generalization (B) phase.

In addition, we did not find significant difference in reaction time (RT) among all groups in either acquisition or generalization phases (Figure 5, all p's>0.12). This is perhaps because the task allowed subjects unlimited time to respond on each trial, and thus there was no time constraints. Accordingly, we believe that our task does not assess speed vs. accuracy measures. In order to assess this measure, tasks should include limitation on response time allowed for subjects [78]. Importantly, most learning and decision making studies usually report number of errors during acquisition.

### Effects of severity of mild cognitive decline on cognitive and homocysteine measures

Given that we found no difference among the control and MCI groups in learning and generalization, we reasoned that severity of MCI symptoms as measured by GDS might reveal differences in learning and generalization. Here, we have conducted further statistical analysis after dividing the MCI group into two subgroups: those with GDS = 2 (very mild cognitive decline, vMCD), and those with GDS = 3 (mild cognitive decline, MCD). As before, the dependent variables included number of errors in the learning and generalization phases, and homocysteine levels.

**Figure 6:**
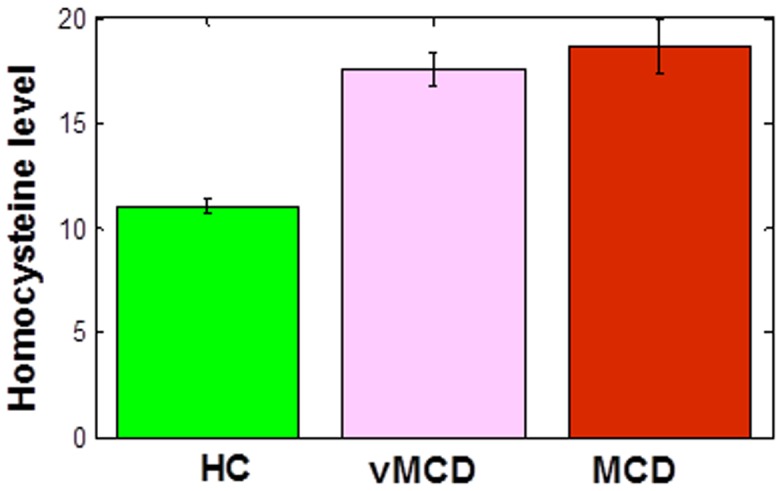
Homocysteine levels in MCI subgroups and healthy controls. Unlike what we expected, there was no effect of MCI severity (that is, between vMCD and MCD) on total homocysteine levels. As before, homocysteine levels in both MCI subgroups are higher than in healthy controls (HC).

**Figure 7:**
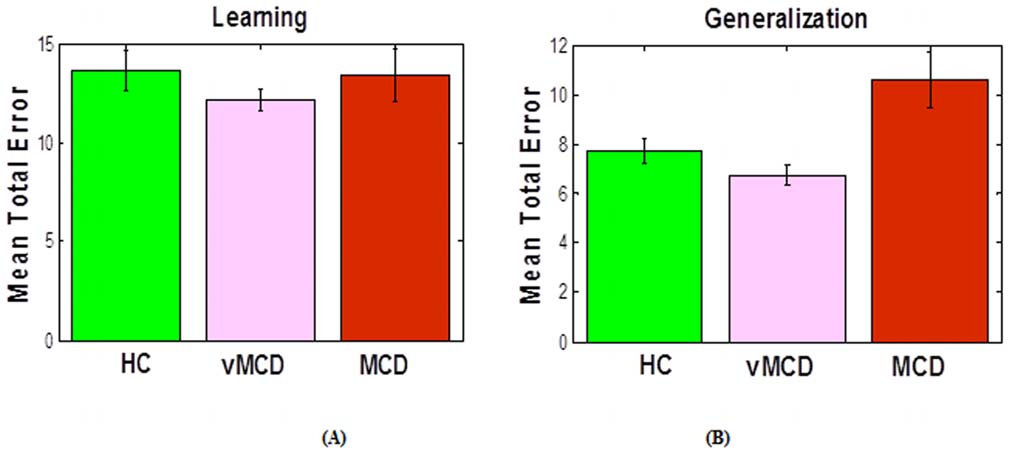
Learning and generalization performance in MCI subgroups and healthy control individuals. (A) The healthy control (HC), vMCD, and MCD groups made similar number of errors during learning; (B) however, on generalization, the MCD group made more generalization errors than controls and individuals with vMCD.

First, we found no difference among the MCI groups (vMCD vs. MCD) in age or education level (p>0.1). In addition, we found that both vMCD and MCD groups have larger homocysteine levels than controls (p <0.03 & p < 0.02, respectively). Unlike what we expected, there was no effect of MCI severity (based on GDS rating) on homocysteine levels (p>0.1, Figure 6). As for cognitive performance, there was no effect of group or MCI severity on the number of errors in the learning phases (Figure 7A). Interestingly, we found that individuals with MCD made significantly more errors in the generalization phase than either individuals with vMCD (p < 0.01) or healthy controls (p < 0.02) (see Figure 7B). There was no difference between vMCD and healthy individuals in the number of errors in the generalization phase (p>0.2).

**Figure 8:**
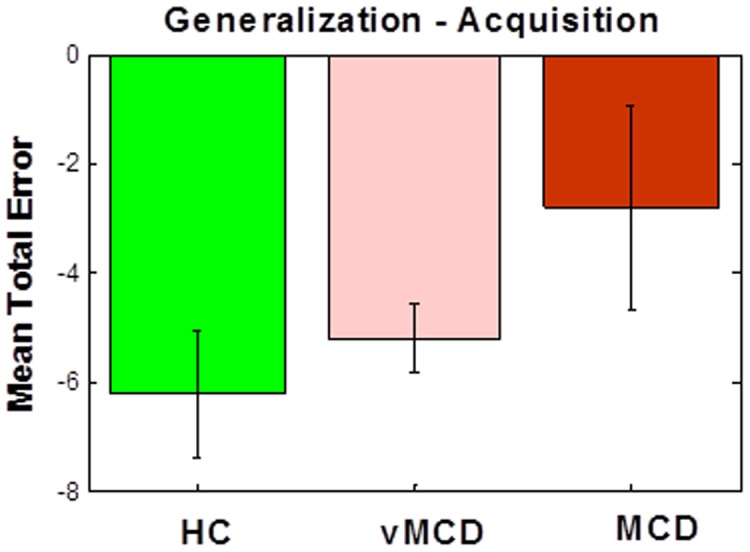
The subtraction of number of errors in learning from generalization (generalization – learning) in MCI subgroups and healthy control individuals. In almost all subjects, this measure is negative since subjects tend to make more errors in the learning than in the generalization phase. Interestingly, the generalization-learning performance in the MCD group were less negative than in vMCD or healthy controls (p < 0.03). Less negative values in the generalization-learning measure stem from comparable performance in learning and generalization. In the MCD group, less negative generalization-learning measure is due to a high number of errors in the generalization phase.

To rule out the possibility that learning phase performance affects generalization phase performance, we subtracted of number of errors in the learning phase from the number of errors in the generalization phase (generalization – learning) for each subject in the MCI subgroups and healthy control individuals. Here, we found that the generalization-learning performance in the MCD group were less negative than in vMCD or healthy controls (p < 0.03; Figure 8). Less negative values in the generalization-learning measure stem from comparable performance in learning and generalization. In the MCD group, less negative generalization-learning measure is due to a high number of errors in the generalization phase.

### Correlations of homocysteine levels and learning and generalization of rules

**Figure 9:**
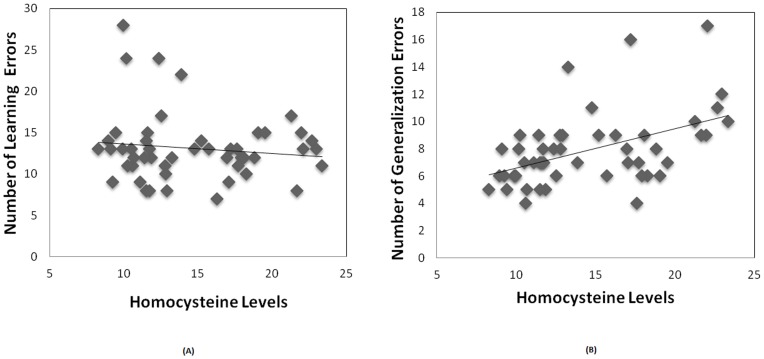
Total homocysteine levels correlate with the number of errors in the generalization phase (B), but not with the number of errors in the learning phase (A).

**Figure 10:**
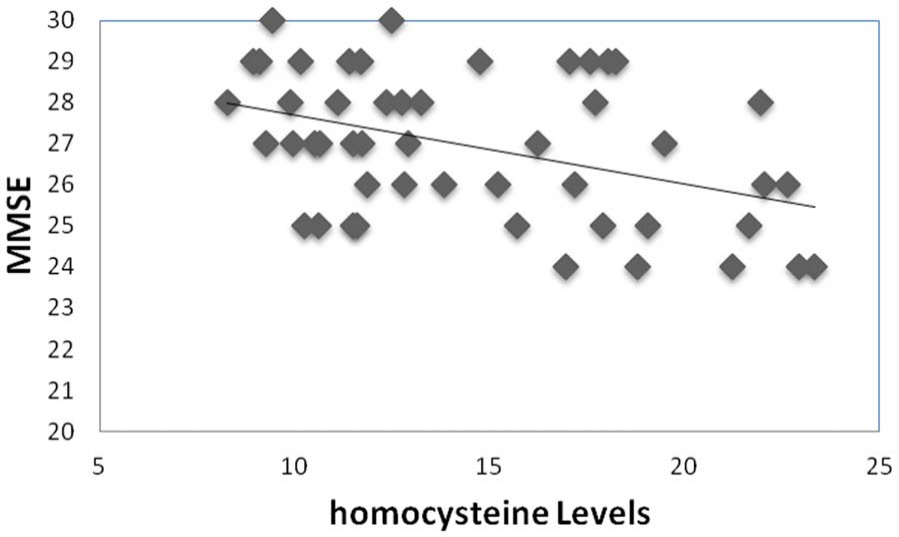
Homocysteine levels inversely correlate with MMSE scores.

Lastly, we conducted correlational analyses between number of errors in the learning and generalization phases and homocysteine levels. As predicted, we found no significant correlation between homocysteine levels and learning performance (Figure 9A, r = −0.12, p>0.3). In contrast, we found a significant positive correlation between homocysteine levels and generalization errors (Figure 9B, r = 0.482, p < 0.001). We also found a negative correlation between homocysteine levels and MMSE scores (Figure 10, r = −0.414, P <0.002).

Give that there was an effect of group on generalization performance, we additionally conducted a multiple regression analysis, with number of errors in the generalization phase as the dependent variable, and homocysteine levels, GDS and CDR as predictors. The overall regression was significant (*p*<.0005, R^2^ = 0.547). Controlling for the GDS and CDR levels, the effect of homocysteine levels on the number of errors in the generalization phase was significant (B = 1.25, p = .005). In addition, once the other factors were controlled, there were no significant effects of GDS or CDR, and no interactions (all p's>0.1).

## Discussion

To our knowledge, this is the first study to test the relationship between homocysteine levels and learning/generalization of rules in healthy subjects and individuals with MCI. Most prior studies that have shown homocysteine levels to be correlated with cognitive performance have used questionnaires, and thus could not assess learning performance [12].

Our results show that homocysteine levels are higher in individuals with MCI than in healthy controls. Using a computerized learning-and-generalization task to test for striatal and hippocampal function, we found no difference in learning and generalization performance in healthy controls and individuals with MCI. We have divided MCI individuals (CDR = 0.5) into two groups, based on the severity of their symptoms, as measured by the GDS. All of our MCI subjects have GDS of 2 or 3. We have found that individuals with MCD made more errors in the generalization phase than individuals with vMCD. There was no difference between the MCI subgroups on the learning phase of the task. Unlike what we expected, we found that homocysteine levels are not significantly different in both MCI groups. Finally, we found that there is a positive correlation between homocysteine levels and generalization errors. We found no correlation between homocysteine levels and learning performance. Our data suggest that dividing individuals with MCI based on measures such as GDS can be informative in terms of their cognitive deterioration and potentially hippocampal dysfunction. We also suggest that hippocampal dysfunction might be stronger in individuals with MCD than in individuals with vMCD. Importantly, our data also show that plasma blood measures can be indicative of cognitive function in individuals with MCI.

The finding that there was no differential effects among individuals with vMCD and healthy individuals suggest that signs of cognitive impairment in MCI might perhaps appear in more severe cases only. One implication of our results is that although individuals with vMCD show no impairment on the cognitive learning-and-generalization tasks (as compared to healthy controls), they have a homocysteine profile that looks like individuals with MCD. This perhaps suggest that homocysteine levels can be a biological marker for MCI in that it can differentiate between healthy controls and individuals with vMCD.

Along the same lines, prior research has suggested that AD involves neural dysfunction that begins before onset of symptoms. It is possible, in our study, that individuals with MCD, were in the prodromal stages of AD. A follow up of these people on clinical and neuropsychological measures would help to verify whether they have prodromal AD.

### Neural substrates of generalization, homocysteine effect, and MCI

In this section, we discuss studies that address the neural substrates (including the hippocampus and prefrontal cortex) of generalization performance, homocysteine effect, and MCI.

Our results are in agreement with prior results showing that hippocampal atrophy is associated with impaired performance in the generalization phase of our task [62]. In a prior study, we also found some older healthy individuals (with potential hippocampal dysfunction) show impaired performance in the generalization phase [79]. Our results are also in agreement with a wealth of studies reporting impaired hippocampal-based cognitive performance in individuals with MCI [80], [81], [82], [83], [84].

Extensive literature has linked generalization performance to the function of the hippocampus, using various behavioral paradigms including transitive inference, generalization of learned rules, sensory preconditioning, and acquired equivalence [57], [58], [59], [60], [62], [65]. In addition, recent studies have also shown a relationship between the hippocampus and generalization of rules in language learning [85]. Importantly, computational modeling and theoretical studies also explain how the hippocampus might mediate generalization processes [86], [87], [88].

Unlike the hippocampus (which plays a role in generalization), some studies report that the prefrontal cortex plays a role in both learning and generalization [see for example 89]. It is possible that the prefrontal cortex participates in the maintenance of rules in working memory, processes that might explain its function in both learning and generalization of rules. For example, recently, Collins and Frank [90] found that working memory plays an important role in rule learning, although we are not aware that establishes a link between working memory and generalization performance.

Similarly, most existing studies found that variations in homocysteine level affect the hippocampus [37], [38], [39]. However, some studies also found that homocysteine acts on the cortex [39], and that hyperhomocysteinemia lead to atrophy in the prefrontal cortex [41]. These studies suggest that homocysteine has a complex effect on the brain. It is not clear whether homocysteine effect on the prefrontal cortex has any relationship to our behavioral results. However, given prior results on the same task, we assume that homocysteine effects on the hippocampus are responsible for the differences in generalization performance in healthy and MCI subjects.

Along the same lines, MCI might have a more complex effect on the brain than assumed here. In the current study, we focused on the effect of MCI on the hippocampus, which is in agreement with an extensive body of literature. For example, individuals with MCI who show hippocampal atrophy on structural imaging are at heightened risk for incipient cognitive decline and AD, relative to nonatrophied subjects [82], [83]. The hippocampus and related medial temporal lobe structures, including entorhinal cortex, show pathology very early in the course of AD [81], [84], [91], [92]. Also, individuals with MCI show impairment on hippocampal-based cognitive tasks, including declarative memory [80].

In addition to the hippocampus, studies found that the prefrontal cortex also deteriorates in individuals with MCI and AD [93], [94]. In addition, some empirical studies argue that prefrontal dysfunction in individuals with MCI might be caused by a disconnection from the hippocampus [95].

Based on previous studies linking generalization impairment to the hippocampus [58], [60], [62], [65], [96], it is plausible that our results are more associated with hippocampal rather than prefrontal dysfunction. In addition, our prior theoretical model [88] shows how generalization deficits can stem from a simulated hippocampal dysfunction in individuals with dementia.

Future research should test whether homocysteine affect cognitive processes associated with the prefrontal cortex, and test whether increased homocysteine levels in the prefrontal cortex contribute to cognitive dysfunction in MCI and AD.

### Clinical implications

There are many definitions of MCI in the literature [73], [97], [98], [99]. Two commonly used clinical definitions of MCI in the literature are CDR or GDS measures. Some studies define MCI based on a CDR score of 0.5 [70], [72], while others define MCI based on GDS score of 3 [43], [75]. In our study, we found that defining MCI based on CDR scores allows for variability in GDS ratings. Specifically, we found that some of our MCI subjects (CDR = 0.5) have GDS scores of 2 or 3 [for similar results, see 47]. The opposite was not true: in our study, all subjects with MCD have CDR score of 0.5. Importantly, we also found that subjects with vMCD and MCD show different cognitive performance, particularly in the hippocampal-based generalization phase of our task. According to Flicker et al. [43], subjects with GDS score of 3 are either MCI or mildly demented. Interestingly, Petersen et al [47] have found that subjects classified with MCI using clinical criteria have either GDS of 2 or 3. These Petersen et al. findings are similar to ours in that various clinical definitions of MCI do not always match.

There have been conflicting results on the relationship between MCI and homocysteine levels. Some studies reported elevated homocysteine levels in MCI individuals [8], while others do not report this association [100]. These conflicting findings could perhaps be related to the various ways MCI is identified. In the Kim et al. [8] and Reitz et al. [100] studies, MCI was diagnosed by a consensus of neurologists and clinical tests based on DSM-IV criteria, rather than using CDR or GDS measures.

The implications of our findings are that it is important to take into account the degree of cognitive impairment in individuals with MCI. Future research should address rates of conversion to AD among subgroups of individuals with MCI. Our work suggests that conversion rate might be higher in individuals with MCD than individuals with vMCD. In addition, low levels of homocysteine combined with low GDS rating should be protective against conversion to dementia in individuals with MCI (and perhaps more so in individuals with vMCD than in individuals with MCD). Future work should test this hypothesis.

Our future work includes building a computational neural network model of the hippocampal region and basal ganglia interactions (following earlier models, see for example [88]) to explain (a) how homocysteine is important for cognitive processes (by linking this to homocysteine effects on acetylcholine and synaptic plasticity in the hippocampus). We will use the model to explain how increased homocysteine levels impair cognition in MCI and AD patients. Christie et al. [35] have found chronic exposure to homocysteine in rats impairs synaptic transmission. In our model, we will simulate impaired synaptic transmission by disrupting weights (simulated synapses) connecting nodes (simulated neurons) in the simulated hippocampal region.

Many studies have reported that lowering homocysteine levels enhances memory and cognition in individuals with MCI and AD [20], [101]. Future work at our lab will investigate whether homocysteine-lowering compounds (such as B12 vitamin supplements) has an effect on the learning-and-generalization task in individuals with MCI and AD.

In sum, our study is perhaps among the first to test the relationship between homocysteine levels and learning and generalization of rules in healthy controls and individuals with MCI. We found that individuals with MCD, but not with vMCD, show impairment at the generalization of rules. We also found increased homocysteine levels correlate with increased number of generalization errors. These findings are in agreement with data showing hippocampal dysfunction in MCI.
